# Why don’t segregated Roma do more for their health? An explanatory framework from an ethnographic study in Slovakia

**DOI:** 10.1007/s00038-018-1134-2

**Published:** 2018-06-16

**Authors:** Andrej Belak, Andrea Madarasova Geckova, Jitse P. van Dijk, Sijmen A. Reijneveld

**Affiliations:** 1 Kosice Institute for Society and Health, Faculty of Medicine, P.J. Safarik University, Kosice, Slovakia; 2 Department of Health Psychology, Faculty of Medicine, P.J. Safarik University, Trieda SNP 1, 040 11 Kosice, Slovakia; 3 Department of Community and Occupational Medicine, University Medical Center Groningen, Groningen, The Netherlands; 4 Department of General Anthropology, Faculty of Humanities, Charles University, Prague, Czech Republic; 5 Olomouc University Society and Health Institute, Palacky University Olomouc, Olomouc, Czech Republic

**Keywords:** Slovakia, Roma health, Health inequality, Adherence, Ethnographic study

## Abstract

**Objectives:**

The health status of segregated Roma is poor. To understand why segregated Roma engage in health-endangering practices, we explored their nonadherence to clinical and public health recommendations.

**Methods:**

We examined one segregated Roma settlement of 260 inhabitants in Slovakia. To obtain qualitative data on local-level mechanisms supporting Roma nonadherence, we combined ethnography and systematic interviewing over 10 years. We then performed a qualitative content analysis based on sociological and public health theories.

**Results:**

Our explanatory framework summarizes how the nonadherence of local Roma was supported by an interlocked system of seven mechanisms, controlled by and operating through both local Roma and non-Roma. These regard the Roma situation of poverty, segregation and substandard infrastructure; the Roma socialization into their situation; the Roma-perceived value of Roma alternative practices; the exclusionary non-Roma and self-exclusionary Roma ideologies; the discrimination, racism and dysfunctional support towards Roma by non-Roma; and drawbacks in adherence.

**Conclusions:**

Non-Roma ideologies, internalized by Roma into a racialized ethnic identity through socialization, and drawbacks in adherence might present powerful, yet neglected, mechanisms supporting segregated Roma nonadherence.

**Electronic supplementary material:**

The online version of this article (10.1007/s00038-018-1134-2) contains supplementary material, which is available to authorized users.

## Introduction

The poor health status of segregated Roma represents the steepest and most persistent health inequalities in Central and Eastern Europe (CEE). Roma make up one of the largest ethnically outlined populations in Europe (Crowe 2007; FRAEU and UNDP 2012) with current estimates ranging up to 12 million persons and a presence in most CEE countries. Facing and adapting to an ongoing history of prejudice, discrimination and paternalist remedial policies, substantial proportions of Roma reside in poor segregated communities (FRAEU and UNDP 2012; Stewart 2012). Compared with the general population, these communities are at the lowest levels of education and income and have the highest rates of unemployment (FRAEU and UNDP 2012). Moreover, they carry the greatest burdens of both infectious and non-communicable diseases and have the shortest lifespans (Cook et al. 2013; EUC 2014).

Well exemplifying the situation elsewhere in CEE (Cook et al. 2013), the poor health status of segregated Roma in Slovakia is also maintained through people’s own everyday practices (FRAEU and UNDP 2012). Despite notable exceptions indicating equal or healthier social support (Bobakova et al. 2015; Kolarcik et al. 2012), alcohol and illicit drug use (Babinska et al. 2014; Kolarcik et al. 2010) and sexual behaviours (Halanova et al. 2014), rigorous studies show that overall Slovak segregated Roma engage in riskier health-related practices than the rest of the population. For example, higher levels of smoking (Belak 2013; Jarcuska et al. 2013), an unhealthier diet (Hijova et al. 2014; Krajcovicova-Kudlackova et al. 2004), unhealthier physical activity (Babinska et al. 2014; Kolarcik et al. 2010), the maintenance of riskier material conditions (Filadelfiova and Gerbery 2012; Majdan et al. 2012) and less effective healthcare use (Belak 2013; Jarcuska et al. 2013) are found among these Roma.

The standard socio-epidemiological approach to explore the drivers behind health-related practices yields practically inconclusive results in the case of segregated CEE Roma. Studies focusing on the associations of health-related behaviours with measures of socioeconomic position (SEP) do not allow questions on why many more Roma live at the lower end of the existing SEP gradients or how such positioning results in more adverse health-related practices to be answered (Földes and Covaci 2012; Reijneveld 2010). Expected associations often do not get confirmed here for all the proxies examined—segregated Roma seem to be doing at least some things differently or to different effects compared to low-SEP segments of the general populations (e.g. Geckova et al. 2014; Janevic et al. 2012; Kolarcik et al. 2009; Voko et al. 2009). Such a situation is common with ethnic health inequalities research in general (Dressler 2005; Smith 2000).

Insight into the driving forces behind the everyday health-endangering practices of CEE segregated Roma is lacking. To advance public health understanding of persisting health inequalities, a sociologically informed exploration of local-level drivers via perspectives of the target populations themselves has long been proposed as a promising starting point both in general (Garthwaite et al. 2016; Singer et al. 2016) and with respect to ethnic health inequalities specifically (Dressler et al. 2005). Recently, several studies qualitatively exploring specific CEE Roma health-related practices have confirmed the expected negative influences of poverty, discrimination and racism (e.g. Andreassen et al. 2017; Janevic et al. 2011; Kelly et al. 2004). However, more comprehensive exploratory studies are still missing.

We therefore explored by way of a sociologically informed ethnographic study the local-level mechanisms that support segregated CEE Roma nonadherence to clinical and public health recommendations.

## Methods

### Theory

We used structural-constructivist relational theories of human action as the conceptual framework of our study (Archer 2000; Bourdieu 1998). According to these theories, everyday practices are driven mostly by the actors’ practical reasoning, which is spatiotemporally contingent, partially implicit and subconscious, and significantly shaped by historically evolving structures and social constructions. The structures represent the environmental, social and bodily conditions in which the selected actors operate. The social constructions represent how the actors interpret these conditions. Actors acquire their specific practical reasoning gradually, through the process of socialization. In this process, the actors’ bodies, inner drives, motivations and interpretative repertoires become practically attuned to their specific conditions. Both acting according to any reasoning in practice and specific socialization patterns continue to depend on enabling environments. What specific structures support which specific practices and how they do so can be best examined by exploring related social constructions that the specific actors use, i.e. their related practical reasoning and their socialization in such reasoning.

### Settings and design

This study was part of a larger longitudinal study exploring the social root causes of poor health status of segregated CEE Roma through the case of a segregated settlement in Slovakia. The larger study spanned 2004–2014 and consisted of four methodologically distinct phases combining ethnography (Reeves et al. 2008) and systematic medical-anthropological interviewing (Hausmann-Muela et al. 2003): (1) a socio-graphic survey, aimed at the selection of a single segregated place; (2) ethnography, aimed at gaining close personal access to and extensive primary data regarding the local everyday health-endangering settings and practices; (3) systematic interviewing, aimed at increasing local representativeness of the collected material; and (4) follow-up communication, aimed at obtaining locals’ reflections on preliminary interpretations and obtaining additional data on long-term outcomes. All fieldwork was carried out by the first author. All aspects of the larger study methodology, relevant also for this study, have been reported in more detail elsewhere (Belak et al. 2017). This regards a description of the setting of the Roma in Slovakia, our procedure for selection of the locality and informants, the characteristics of the selected settlement, the observation and elicitation procedures that we used in all phases of the study, our initial coding of the study data, the first author’s embeddedness in the settlement and the study’s potential biases.

In the terms of the theories outlined above, the primary focus of this study was on what local structures supported the actors’ eventual nonadherence to clinical and public health recommendations over the long term (cf. Frohlich et al. 2001; Singer et al. 2016). Drawing on the theories, we started with an exploration of the reasoning of local Roma regarding nonadherence (cf. Cockerham 2005; Frohlich et al. 2001). Then, we explored what experiences contributed to the adoption of such reasoning (cf. Singh-Manoux and Marmot 2005; Williams 1995). Finally, we explored local-level mechanisms that supported both the adoption of such reasoning and the everyday practice of pro-nonadherence reasoning, i.e. the local structures that systematically enabled such adoption and such practices (Hedström and Ylikoski 2010).

### Samples/informants

In phase 1 (July 2004), we selected a single segregated settlement with a growing population of approximately 260 people (230 in 2004, 300 in 2014) on the outskirt of a village with a declining non-Roma population of about 530 (580 in 2004, 470 in 2014). In 2004, approximately half of the Roma settlement’s inhabitants were children under age 15 years, and only 5 people were older than 60. In phase 2 (September 2004–October 2005), we obtained data on approximately 90 people belonging to one of the three then largest extended families in the settlement. In phase 3 (October 2005), we visited a sample of 10 of the settlement’s 48 households. The sample was representative according to the households’ social ranking (low, medium and high), based on the combination of ascribed affluence and prestige, and kinship affiliations (to the three largest extended families). In these households, we interviewed 28 people, 22 of them adult women. Locally, men were considered less competent regarding health-related issues both by themselves and by women, and most of them also showed less interest in discussing health spontaneously. None of the people approached refused to participate in the interviews. Phase 4 follow-up observations and elicitations (November 2005–November 2014) were limited to approximately 15 Roma personally closest to the first author.

### Procedure

The data consisted of field notes on direct observations and written and audio records of elicitations obtained during phases 2–4. We collected observations, spontaneous declarations and replies in elicitations regarding: why individual Roma did not adhere to selected clinical and public health recommendations—as data on reasoning for nonadherence; what experiences individual Roma considered important for their adoption of such pro-nonadherence reasoning—as data on the adoption of pro-nonadherence reasoning; and how and what local circumstances supported the recurrence of such contributing experiences and nonadherence practices—as data on local-level supporting mechanisms.

To gain data specifically and exhaustively on contemporary clinical and public health recommendations, an encyclopaedic practitioner’s handbook covering both clinical and public health knowledge and recommendations was used throughout all phases of the study to guide observations and elicitations in terms of topics (Sasinka et al. 2003).

### Coding, analysis and reporting

For coding the data we re-used transcripts from previous analyses (Belak et al. 2017). In these transcripts, all field notes and audio recordings relevant regarding health-related settings and practices were already merged and coded for relevance in relation to household social levels, kinship affiliations, genders, ages, time periods, and domains of exposures and their core elements, as defined in a widely used eco-social framework on social determinants of health (WHO 2010). The first author then in steps added new axial codes signifying “pro-nonadherence reasoning”, “experiences contributing” to the adoption of such reasoning and local-level “mechanisms”.

Next, we performed a qualitative content analysis using recurrent abstraction (LeCompte and Schensul 2013). We repeatedly read and in steps summarized all text sequences coded as relating to pro-nonadherence reasoning, experiences contributing to the adoption of such reasoning, and local-level mechanisms. We focused on capturing local variability and the most salient kinds, as follows. First, we summarized sequences on local reasoning, yielding 13 kinds of reasoning. We have reported these in Appendix 1 in electronic supplementary material. Second, we summarized sequences of experiences contributing to the adoption of pro-nonadherence reasoning, yielding four such kinds. We have reported these in Appendix 2 in electronic supplementary material. Then, we summarized sequences on local mechanisms, yielding seven mechanisms in total. We report these mechanisms in our results below. During this analysis, we realized that some of the mechanisms were controlled by and operating through Roma actors in the settlement, while others more by local non-Roma actors outside the settlement. We report according to this distinction, as it informs on which actors need to be prioritized in interventions and regarding what mechanisms.

During the analyses, we also realized that the identified mechanisms supported each other. Given that most of the identified individual mechanisms have been previously described with respect to CEE Roma (see “Introduction” section), we focused mainly on reporting their mutual interrelations. For more information and illustrative examples, we refer the reader to Appendices 1, 2 and 3 in electronic supplementary material and to our previously published detailed descriptive report on other parts of the same study (Belak et al. 2017).

## Results

We identified seven local-level mechanisms that supported the adopting of pro-nonadherence reasoning by Roma and their nonadherence practices. Below, for each of these mechanisms we first briefly describe their underlying structure and then list and illustrate how they worked (for further details and illustrations, see also Appendices 1, 2 and 3 in electronic supplementary material). In the last section, we describe how these mechanisms mutually supported each other.

### Mechanisms controlled by and operating through local Roma

#### Roma situation of poverty, segregation and substandard infrastructure

The Roma settlement was trans-generationally extremely poor, segregated both socially and physically from the local non-Roma village and had substandard infrastructure. This directly supported the adopting of pro-nonadherence reasoning by Roma youth, as it contributed to their frequent failures of adherence, and because some aspects of the setting were experienced by the youth as significantly positive. For instance, when trying to adhere to medication advice, young families experienced an inability to cover costs of medications due to low income, to understand clinical recommendations due to substandard education, to preserve documentation on their diseases due to a lack of personal storage space, etc. Yet, to illustrate their perceived positive experience of this setting, young people found that the segregated housing setup (yards shared by extended families outside the non-Roma village) enabled convenient child supervision (no contact with strangers or car traffic, etc.).

The setting directly supported nonadherence practices via a constant lack of means for adherence and a constant availability of means for local alternatives to adherence. Except for the highest ranked families, Roma lacked the income, information and infrastructure necessary to maintain “outside” (i.e. non-Roma) standards of personal healthcare (hygiene, safety measures, healthcare services access, etc.). Meanwhile, the substandard infrastructure and spatial segregation enabled unhealthier ways of provision of, e.g. heating (wooden stoves, proximity of forest), water, electricity (unsafe illegal connections) and waste disposal (unsanctioned garbage piles).

#### Roma self-exclusionary ideology and misinformation

The dominant views in the settlement claimed the general or relative (compared with adherence to local alternatives) inappropriateness for Roma of adherence to alleged outside standards. Such inappropriateness was typically framed in racialized and gendered ethnic terms quoting outdated racist expert concepts. This directly supported Roma youth in adopting pro-nonadherence reasoning by presenting appealing interpretations of standard local experiences with adherence failures, adherence and nonadherence. For example, youth interpreted some of the adherence failures they experienced as being due to their personal incapacities (e.g. by their strong negative feelings regarding most aspects of hospitalization) as results and proofs of “natural Roma/Gypsy” collective bodily incapacity, quoting allegedly specific Roma biology (Roma genes, blood, brains, etc.).

#### Roma socialization for their situation

Based on experiences and dominant local interpretations of them, youth in the settlement gradually adopted practical reasoning that favoured local alternatives over outside standards. Adoption of pro-nonadherence reasoning formed an integral part of this contrastive mode of socialization: youth lacking appropriate means to adhere and being exposed to dominant local self-exclusionary views and misinformation gradually resigned and became adults who lacked the means and motivation needed to adhere. The socialization directly supported local nonadherence practices by generating adults prone to practice and further develop rather local alternatives to adherence regarding their health problems in general.

#### Roma alternative practices

Most adults in the settlements appraised, practiced and developed standards understood as more appropriate alternatives to alleged outside standards. The better appropriateness of such standards for Roma was typically framed in racialized and gendered ethnic terms in line with local self-exclusionary ideologies. Relatively riskier health behaviours and a less attentive approach to their own health formed an integral part of such alternative practices.

This approach directly supported Roma youth in adopting pro-nonadherence reasoning by generating direct negative experiences with pro-adherence experiments and positive experiences with Roma alternatives to adherence. For example, youth witnessed their parents’ frequent failures to adhere to clinical recommendations. Simultaneously, they experienced some aspects of Roma alternatives to clinical recommendations as advantageous (e.g. compared to institutional care, they viewed homecare in the case of treating alcohol dependency as providing comparable health effects and better social side effects; see also Appendix 3 in electronic supplementary material).

### Mechanisms controlled by and operating through local non-Roma

#### Non-Roma anti-Roma ideologies and misinformation

Local non-Roma typically lacked information regarding most aspects of everyday life in the Roma settlement and expressed beliefs that Roma were naturally unable to maintain non-Roma standards. To support the latter, they quoted outdated expert concepts and personal experiences with deliberate Roma nonadherence practices. Such perspectives mostly supported Roma youth and early adolescents in adopting pro-nonadherence reasoning indirectly, by inspiring Roma self-exclusionary ideologies. Such views also indirectly supported Roma alternative practices via consequent non-Roma discrimination, racism and dysfunctional support for Roma (see below).

#### Non-Roma discrimination, racism and dysfunctional support towards Roma

Local non-Roma often acted towards the Roma in a discriminatory and racist manner. Moreover, even sincere local non-Roma attempt to provide support to Roma, typically drawn implicitly on racist or otherwise misinformed concepts, usually lacked practical functionality. Such approaches directly supported the adopting of pro-nonadherence reasoning by Roma youth by contributing to their experiences of related adherence failures. Examples of discrimination are that the Roma youth experienced longer waiting times as well as racist slurs from personnel. An example of dysfunctional support is that a public hygienic centre was installed in the settlement by the local municipality without consulting the local Roma. It then went ignored by the Roma community because it was impractical and ugly.

#### Drawbacks in adherence

The restrictive aspects of clinical and public health recommendations were mostly considered by the Roma as inherently conflicting with a “good life”, typically framed in racialized and gendered ethnic terms in line with the dominant local Roma self-exclusionary ideologies and misinformation. Such aspects directly supported Roma youth in adopting pro-nonadherence reasoning by labelling adherence as disadvantageous. For example, Roma youth found some clinically successful experiments with adherence (e.g. proper management of chronic diseases via dietary restrictions) to lead to too significant losses in terms of the quality of life that “real Roma” could and should prefer.

### An interlocked system of local-level mechanisms

**Fig. 1:**
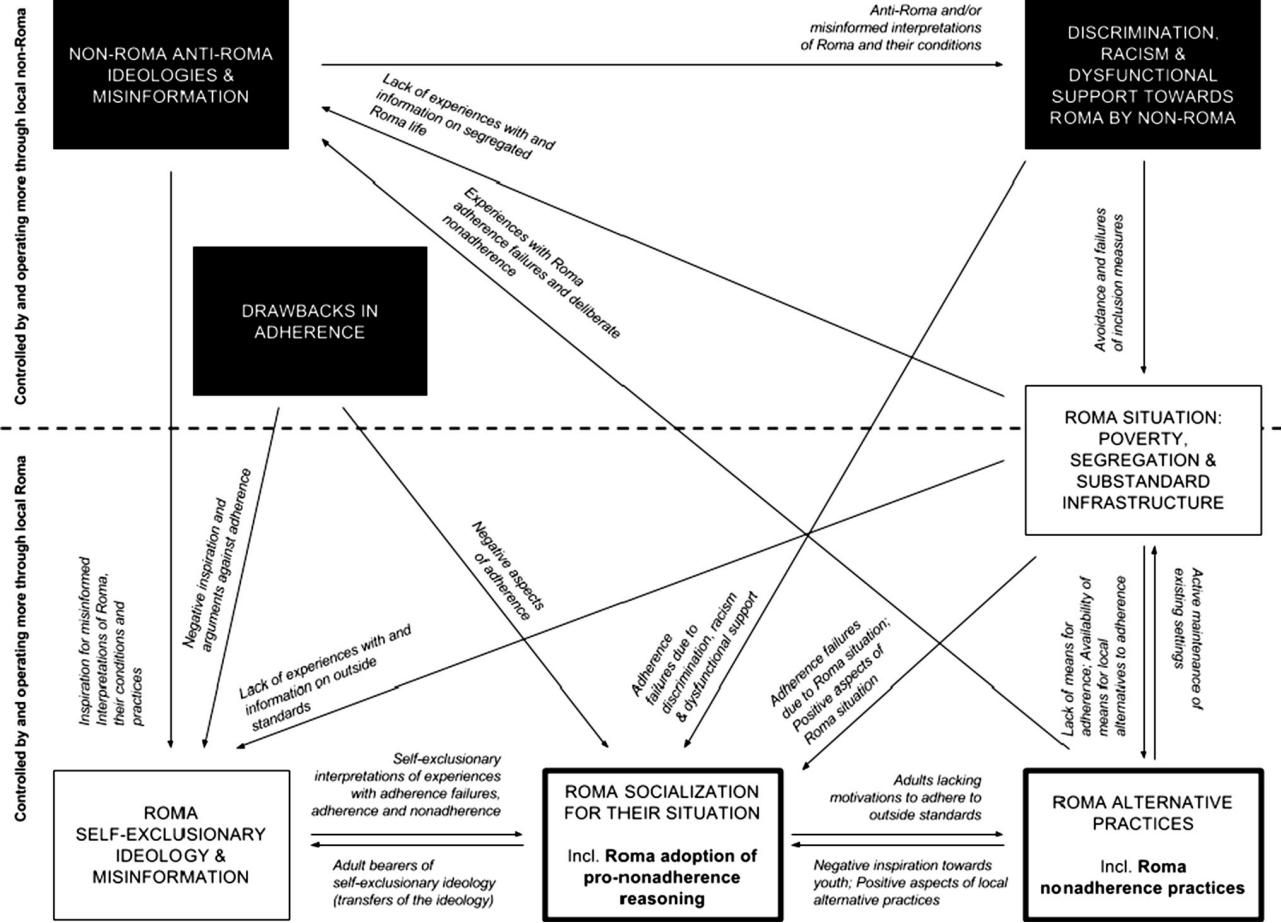
Local-level mechanisms supporting segregated Roma nonadherence reasoning and practices, Slovakia, 2004–2014

The identified mechanisms formed an interlocked system, as schematically summarized in Fig. 1.

#### Mechanisms controlled by and operating through local Roma

The Roma situation of poverty, segregation and substandard infrastructure directly supported Roma self-exclusionary ideologies and misinformation by generating a lack of experience with and information on outside standards. For example, the Roma were unaware that racist expert theories about Roma “natural incapacities” were outdated. They further lacked experience in how under different circumstances some adherence practices can become compatible with high-quality life according to their criteria. This setting also directly supported non-Roma anti-Roma ideologies and misinformation. For instance, due to segregation, non-Roma locals lacked experience and information on the efforts that Roma had to make regarding personal healthcare and the constraints that they faced.

Roma socialization for their situation directly supported the Roma self-exclusionary ideology and misinformation by raising adults who contributed further to the trans-generational transfer of such perspectives. For example, adults would mock and ridicule youth experiments with adherence as “too non-Roma like”, “unnatural for Roma”, “too feminine”, etc.

Roma alternative practices directly supported the Roma situation of poverty, segregation and substandard infrastructure, as they included the active maintenance of existing settings. For instance, adult Roma kept investing in and repairing the substandard local infrastructure. Roma alternative practices also directly supported the non-Roma in maintaining their anti-Roma ideologies and misinformation, as the non-Roma frequently observed deliberate Roma failures to adhere (e.g. apparently deliberate Roma withdrawals from life-saving clinical plans).

#### Mechanisms controlled by and operating through local non-Roma

Anti-Roma ideologies and misinformation among non-Roma directly inspired the self-exclusionary ideologies of Roma. For instance, in their own racialized explanations of Roma nonadherence, Roma would often quote the racist “expert” views of non-Roma. Such perspectives also directly supported non-Roma discrimination, racism and dysfunctional support, as they provided a seemingly reasonable rationale for such practices. For example, local non-Roma professionals would often quote misinformed knowledge (e.g. assumed Roma social norms that do not exist) or racist assumptions (i.e. assumed natural Roma incapacities) when justifying their own standard of not consulting the Roma themselves. In turn, this non-Roma discrimination, racism and dysfunctional support directly contributed to the Roma situation of poverty, segregation and substandard infrastructure (e.g. municipal representatives neglected the maintenance of community infrastructure within the settlement).

The drawbacks in adherence directly supported the Roma self-exclusionary ideology and misinformation by presenting arguments against adherence. For instance, Roma proponents of nonadherence to outside standards would often quote their personal negative experiences with restrictive aspects of adherence as their reasons for nonadherence (e.g. detachment from family during hospitalization).

## Discussion

In our study, we explored local-level mechanisms supporting segregated Roma nonadherence to clinical and public health recommendations. We identified seven such mechanisms: the Roma situation of poverty, segregation and substandard infrastructure; Roma socialization into their situation; the perceived value of Roma alternative practices; exclusionary non-Roma and self-exclusionary Roma ideologies; discrimination, racism and dysfunctional support towards Roma by non-Roma; and drawbacks in adherence. We found that these mechanisms formed an interlocked system controlled by and operating through both local Roma and non-Roma.

We found Roma nonadherence practices were being directly supported by non-Roma practices of discrimination, racism and dysfunctional support as well as by the Roma situation of long-term multi-dimensional segregation. This matches previous research well. Janevic et al. (2011) distinguished three levels of racism in a study on Roma women’s access to prenatal and maternity care in Serbia and Macedonia: internalized racism, personally mediated racism and institutionalized racism. Most of the processes through which they exemplified these levels are corroborated by our findings and examples, as well. Our findings thus confirm that both the current and historical non-Roma discriminatory approach to Roma still very much negatively affects CEE Roma adherence to clinical and public health recommendations.

We found that discrimination and segregation steadily generated frustrating experiences for Roma youth, which significantly contributed to the formation of adult Roma identities leaning towards deliberate and proud development of practices alternative to adherence. Previous research on the negative effects of discrimination and segregation on health-related practices has typically focused on processes working as direct everyday constraints to healthier behaviours, regarded as such also by members of the negatively affected minorities themselves (cf. Bailey et al. 2017; Janevic et al. 2011). Our findings show how non-Roma discrimination and Roma multi-dimensional segregation might significantly support Roma nonadherence practices not only as direct barriers but also through shaping the identities of Roma with respect to health. This finding also offers an intelligible non-racist explanation for the non-Roma neighbours’ common experiences of deliberate nonadherence of segregated Roma, even in the absence of any apparent imposed constraints.

For our Roma informants, racist and racialized ideologies appeared to serve as direct inspirations for the reasoning they used to explain their negative experiences with adherence. In current health-research approaches (cf. Bailey et al. 2017; Janevic et al. 2011), such ideologies, i.e. rendering minority people naturally less capable of adherence, are usually understood as adverse to health inequalities, because they shape existing and have shaped majorities’ practices towards minorities. Our findings show how local Roma youth, socialized under the influence of such ideologies, tended gradually to become adults understanding themselves as naturally less competent and more likely to fail at adherence. Social scientific research of racial domination has long recognized similar vicious circles of racialized self-fulfilling prophecies as being behind practices supporting ethnic inequalities (e.g. Fanon 2008; Fassin 2011) and was also recently summarized by Grill (2017) directly for Slovakia. Our findings thus show how along with supporting non-Roma discrimination of Roma, racist and racialized ideologies might also support Roma nonadherence through the process of Roma socialization.

We also found that our informants’ nonadherence practices were supported by the perceived drawbacks in adherence. In research on health inequalities as well as in related interventions, clinical and public health recommendations are usually understood and used as standards which most people familiar with their functionality and possessing means for adherence to them consider as appropriate. However, in-depth qualitative research often finds people with good understanding and sufficient means still resisting such standards for a great variety of other reasons—including the view they possess better alternatives (Merrild et al. 2017; Trostle 2004). In their own view, our informants were sometimes capable of coming up with alternative practices, leading to outcomes of possibly comparable health effects and more positive social side effects. This finding supports a view common in other ethnographies of segregated Roma and analogous groups (e.g. Stewart 1997; Tauber 2006; Williams 2003): that these groups sometimes carve out socio-material niches for themselves that enable the development of genuine lifestyle alternatives that cannot be downplayed by outside standards as mere rhetoric or as something only segregated Roma can experience as valuable due to their previous socialization into segregation.

### Strengths and limitations

The main strength of our study is its sociologically well-theorized and applied approach. The use of ethnographic methods enabled intimate access to local everyday settings, local people and their practical reasoning. The systematic interviewing across several local stratifications allowed for local representativeness as well as for topical omissions in the previous less-systematic phase to be considered. The follow-up communication enabled additional reflections of preliminary interpretations by local core informants. Choosing structural-constructivist sociological theories and the WHO framework on social determinants of health (WHO 2010) strengthens both the sociological and public health significance of our results.

Our research design and our reporting also had some limitations. First, the fieldwork, coding and most of the analyses were performed by a single researcher, limiting the potential for inter-personal corroboration. However, this is standard in ethnographic research, given the logistic difficulties connected with embedded research. Second, the researcher conducting the fieldwork was a male, which may have influenced the reporting by women due to existing local gender power-imbalances. This may have resulted in, e.g. underreporting of gender-sensitive issues. However, we think that this bias is rather limited, as the first author also experienced numerous intimate conversations with local women across generations which included strong criticisms of local male and female roles. Third, it was impossible to remain personally embedded in the settlement during the full study-period. Nevertheless, throughout the research period the first author did stay personally very close to members of one of the three major local extended families which he kept visiting. Fourth, given the author’s embeddedness within the Roma settlement, data on local non-Roma views and practices were much more mediated compared to the data on local Roma. However, all assertions implying non-Roma social norms or practices were still based only on real and documented cases. Despite all the reassuring circumstances just mentioned and the above discussed general good match of our findings with other published research, the presented framework cannot be generalized.

### Implications

Future research and interventions aiming at behavioural changes of segregated Roma towards adherence to biomedical recommendations should include discussions with the Roma about whether some of their identity-related preferences for nonadherence might not present a case of symbolic violence (Bourdieu 2000), i.e. values historically imposed on their communities by powerful non-Roma. Second, interventions need to address the local anti-Roma prejudices and malpractices of local non-Roma, as well as the influences of more distal actors and processes that support such local mechanisms (e.g. poor education and media coverage in CEE regarding racism and Roma history and conditions). Third, the research and interventions should always carefully examine the health-related outcomes and social side effects of eventual alternative Roma care practices.

### Conclusions

Segregated Roma might be doing less for their health due to interlocked systems of local-level mechanisms, controlled by and operating through both local Roma and non-Roma. Racist non-Roma ideologies, internalized by Roma into a racialized ethnic identity through socialization, and drawbacks in adherence might represent powerful, yet neglected, mechanisms supporting segregated Roma nonadherence.

## Electronic supplementary material

Below is the link to the electronic supplementary material.

Supplementary material 1 (PDF 83 kb)
