# Supplementary material for: Why don’t segregated Roma do more for their health? An explanatory framework from an ethnographic study in Slovakia
Source: Int J Public Health. 2018 Jun 16;63(9):1123–31. doi: 10.1007/s00038-018-1134-2 (PMC6245243; doi:10.1007/s00038-018-1134-2)
Supplement: Supplementary file 1 — Supplementary material 1 (PDF 83 kb) [file 38_2018_1134_MOESM1_ESM.pdf]

International Journal of Public health

Why don't segregated Roma do more for their health? An explanatory framework from an ethnographic study in Slovakia

Andrej Belak MSc[1,2,3,4], Prof Andrea Madarasova Geckova PhD[1,2,5], Assoc. Prof Jitse P. van Dijk MD PhD[1,3,5], Prof Sijmen A. Reijneveld MD PhD[3]

Affiliations:

1 Kosice Institute for Society and Health, Faculty of Medicine, P.J. Safarik University Kosice, Slovakia

2 Department of Health Psychology, Faculty of Medicine, P.J. Safarik University Kosice, Slovakia

3 Department of Community and Occupational Medicine, University Medical Center Groningen University of Groningen, Groningen, the Netherlands

4 Department of General Anthropology, Faculty of Humanities, Charles University, Prague, Czech Republic

5 Olomouc University Society and Health Institute, Palacky University Olomouc, Olomouc, Czech Republic

Correspondence:

Andrej Belak, MSc

Department of Health Psychology

Faculty of Medicine, P.J. Safarik University

Trieda SNP 1, 040 11, Kosice, Slovakia

Phone: +421 55 234 3392

E-Mail: andrej.belak@upjs.sk

Appendix 1 Kinds of reasons the Roma had for nonadherence to clinical and public health recommendations

We identified 13 kinds of reasons. During the analysis, we realized that some of the reasoning was framed and explained by the local Roma informants as structural constraints: “I would like to adhere to this, but I cannot, because here we lack…”, while the remaining reasoning was framed and explained as individual preferences: “I could adhere to this, but I don’t want to, because I prefer…”. In addition, in the second case, the preferences were typically accompanied by spontaneous expressions also in collective-identity terms (“Here, we / Roma prefer…”). Below, we report according to this distinction, as it informs where cooperation of the targeted Roma in eventual interventions might be anticipated more readily (the perceived structural barriers), where not (the preferences) and why, according to the target population itself. After a brief basic description, by each kind we follow with: an estimate of its variability and validity, i.e. how occurrence of such reasoning varied across local strata and how the declared reasoning differed from actual practice in some cases; public health relevance, i.e. for which exposure domains and elements this kind of pro-nonadherence reasoning was potentially relevant; and illustrative examples.

Kind of reason	Description	Variability and validity	Public health relevance	Examples
		Kinds of reasons framed as imposed lack of means		
**Lack of knowledge**	Clinical or public health recommendations or healthcare services information not known or not practically understood	More common with decreasing social status and in men	All exposure domains	-Unknown nutritional requirements for newborns, certain diseases and diseases processes, smoke toxicity, etc.<br>-Poor practical understanding of recommended medication regimens<br>-Poor understanding of hospitalization requirements
**Lack of funds and sustenance opportunities**	No savings, monthly insolvency regarding most basic needs, no legal employment and long-term income insecurity	Not common only in several highest-ranked households in some periods; more intense with decreasing social status	All exposure domains	-Highest-ranked households sometimes could not afford transportation costs related to management of chronic diseases<br>-Lowest-ranked household members could rarely afford prescribed medicines requiring supplementary payments
**Inadequate infrastructure**	Missing or dysfunctional basic public and household infrastructure and amenities	Somewhat less frequent in several highest-ranked households; more severe with decreasing social status	Material circumstances (incl. environmental exposures), community and personal hygiene	-Single highest-ranked household possessed a bathroom and laundry machine with majority of households not possessing any water taps<br>-All households used provisional dry toilets, wood stoves for both heating and cooking, and many only possessed provisional illegal connections to electricity<br>-There was no public lighting, sewerage system and functional waste-disposal scheme in the settlement
**Lack of self-esteem**	Hesitance regarding social transactions with non-Roma outside the local village due to stigmatization, lack of knowledge and lack of communication skills	Somewhat less frequent only in several highest-ranked households; greater with decreasing social status	Healthcare use; linking and bridging of social capital	-Even the highest-ranked people often hesitated regarding, chose not to take part in, or opted out of recommended clinical scenarios halfway, quoting “too much” of “too confusing and embarrassing dealing” with healthcare service provider staff, etc.

Kind of reason	Description	Variability and validity	Public health relevance	Examples
**Lack of communication skills**	Communication problems due to own lesser competence in Slovak, esp. in technical terminology and standard communication styles, and general non-Roma incompetence in Romani*	Somewhat less frequent only in several highest-ranked households; more severe with decreasing social status	Healthcare use; linking and bridging of social capital	-Even highest-ranked people had difficulties understanding their diagnoses and related clinical recommendations unless carefully explained at a relatively slow pace and translated into lay Slovak<br>-Single local clinical practitioner occasionally attempted use of single Romani words upon trouble with medical interviews (anamneses)
**Lack of means to prevent mistreatment by non-Roma**	Unavailability of effective procedures to ensure one will not be treated impolitely, harshly or offensively once identified as a Roma	Somewhat less frequent only in several highest-ranked households; greater with decreasing social status	Healthcare use; linking and bridging of social capital	-Some people, typically the highest ranked, were better equipped (better clothes, better knowledge and communication skills, lighter skin) for strategic concealment of their Roma origin and for effective negotiation of fairer treatment (e.g. through polite expressions of loyalty)<br>-Most other people felt avoidance of individuals with a racist or discriminatory track-record was the only effective strategy of prevention against own mistreatment within the healthcare services
**Lack of trust**	Doubting functionality of clinical and public health recommendations and competence or intentions of healthcare practitioners	Only occasional across social levels (typically based on incidental individual negative experiences or on rumours of the same)	All exposure domains	-Doubts regarding functionality of dietary recommendations with respect to chronic diseases<br>-Doubts regarding safety of narcosis<br>-Conviction of the incompetence of an allegedly alcohol-dependent paediatrician<br>-Suspicion of bad intentions by overtly anti-Roma practitioners
Kinds of reasons framed as personal / Roma lack of motivation				
**Moral preferences**	Inappropriateness of adherence to recommendations expressed in racialized and gendered ethnic terms; general or relative in comparison to local alternatives	Common in all strata as rhetoric; in practice, not followed as strictly or at all with respect to children and with increasing social status	All exposures	-For adult men, attentiveness to one's own health and careful adherence to clinical and public health recommendations was considered "too non-Roma-like" (*gadžikano*) and "too feminine"; women caring for everybody's health was by everybody considered "naturally" more appropriate "certainly, by Roma"<br>-Highest-ranked adult women kept experimenting with adherence to recommendations (e.g. regarding management of chronic diseases and exercise), but often in concealment due to embarrassment and arguing by dropouts with relief, they were "not that non-Roma-like after all, despite being snobby" (*gizdave*)<br>-Regarding health problems in children and elderly, especially in acute cases, wherever required means were

Kind of reason	Description	Variability and validity	Public health relevance	Examples
				available most adults tried to ensure adherence to clinical recommendations
**Aesthetic and sensory preferences**	Inappropriateness of adherence to recommendations expressed in aesthetic or sensory terms; often accompanied by statements of this being an ethnic trait; general or relative in comparison to local alternatives	Equally common across local stratifications	All exposures	-Any recommended practices or serious discourses concerning one's bodily functions, especially regarding lower half of the body (e.g. personal hygiene, treatment of health problems, including genitalia or feet and condom use) were regarded as highly "disgusting and embarrassing for Roma" and as such remained contained within the individual's most private sphere and barely practiced (e.g. secrecy and hesitance regarding installation and maintenance of bathrooms and toilets, preventive gynaecological check-ups, sharing of related knowledge)<br>-Other recommendations-related phenomena, similarly avoided as "disgusting", "unattractive" or "unbearable" in contrast to local alternatives, included e.g. most healthy foods, work-related safety measures; silent, solitary living-spaces and places where people have died (e.g. hospitals)
**Effectivity of local social norms, public control and violence**	Conviction that local public rules, surveillance, peer pressure and socially accepted patterns of physical violence (i.e. among partners and towards children) presented sufficient measures to prevent socially unacceptable health-related practices	Equally common across local stratifications	Promiscuity, birth-control, all exposures by children	-Adherence to the social norm of unacceptability of unmarried girls and women spending any time alone with unrelated adult men was being carefully monitored by the whole public, breaches were strictly sanctioned by ostentatious partner violence, and this practice was considered and praised by both men and women as "appropriate and effective" also privately<br>-Strict adherence of children to any procedures considered appropriate by adults – including clinical recommendations – was imposed by adults as a must, sometimes using threats of physical violence or violence, and this practice was considered more reasonable and effective than negotiations of the children's understanding and content
**Pride regarding and preferential valuation of local knowledge, talents and settings**	Preference for the development and use of local practices, techniques and settings considered alternative to those recommended (often even where the latter were understood as more efficient, effective and cheaper), framed as a deliberate expression and	Common in all strata as rhetoric; in practice, followed somewhat more often with decreasing social status	Healthcare use; material circumstance; and risky behaviours	-High and low-ranked people alike praised therapeutic procedures from folk herbal medicine for their efficiency and efficacy, but the former practiced it less in reality, favouring medical treatment instead<br>-With decreasing knowledge of and access to medical procedures along with decreasing social status, pioneering experimental therapeutic practice was more common and praised (e.g. petrol therapy for syphilis)

Kind of reason	Description	Variability and validity	Public health relevance	Examples
	proof of local independence and virtuosity; often accompanied by statements of this being an ethnic trait			-While understood as relatively inefficient and risky compared to outside standards, locally specific infrastructural arrangements (e.g. illegal constructions from reclaimed industrial materials) were considered sufficient and preferable as they enabled development and display of "typical Roma practical ingenuity" and "freedom"<br>-The local housing setup was supposed to enable better shared public oversight of children (and their safety) by adult members of extended families
**Coping with stress**	Deliberate practices of leisure activities understood as health-endangering in the long-term, yet effective and indispensable for handling of psychosocial pressures faced; often accompanied by statements of their ethnic specificity	Equally common across local stratifications	Risky behaviours	-People commonly expressed that they considered daily smoking, occasional binge drinking, occasional opulent unhealthy foods and occasional gambling necessary to maintain their "sanity" due to the relative harshness of their condition (compared to non-Roma standards) vis-à-vis "natural Roma susceptibility" to pain, discomfort, stress, sadness, etc.
**Solidarity with non-Roma**	Deliberate minimalization of contacts with non-Roma based on the proposition that non-Roma appropriately experience contact with segregated Roma as difficult	Invoked occasionally, equally frequently across local stratifications	Healthcare use; linking and bridging social capital	-Upon deciding between different clinical scenarios (e.g. regarding hospitalization) or discussing risks of mistreatment in clinical settings, the Roma also commonly expressed and considered pity for non-Roma staff having to endure "the Roma ways they are not used to, don't understand and cannot stand" (e.g. loud large-groups visits)

*While most local Roma understood and spoke Slovak fluently, their mother language was Romani, i.e. a language closest to modern Hindu (Matras 2002)

Matras Y (2002) Romani: a linguistic introduction. Cambridge University Press, Cambridge

Appendix 2 Experiences contributing to adoption of pro-nonadherence reasoning

1) Adherence failures due to imposed constraints

This kind regarded frustrating experiences of young Roma, especially in their late teens and their twenties, willing and attempting to adhere to selected clinical and public health recommendations but finding themselves unable to do so due to constraints perceived as not controlled by the local community. These constraints included local non-Roma racism, discrimination, dysfunctional support towards the Roma, and the Roma situation of poverty, segregation and substandard infrastructure. E.g. young people initially willing and attempting to investigate their health problems gradually developed a feeling of lack of means to do so due to discrimination and racism after experiencing recurrent refusals of services and mistreatment by healthcare staff directly quoting or implying Roma ethnicity. Young families' indebtedness due to transportation and complimentary medication costs connected with management of chronic diseases and their inability to preserve medical documentation due to lack of personal storage space represent common examples of frustrating experiences with adherence failures, understood as being due to dysfunctional support by non-Roma and to the Roma situation of poverty, segregation and substandard infrastructure.

2) Adherence failures due to personal/Roma incapacities

This kind regarded frustrating experiences of young Roma, especially in their teens and twenties, who were willing and attempting to adhere to selected recommendations but found themselves unable to do so due to perceived personal weaknesses. Typically, such experiences were also interpreted by the Roma as collective traits framed in racialized and gendered ethnic terms. E.g. after several unsuccessful attempts, persons unable to cease smoking gradually started feeling as not being able to do so due to their personal inherent weaknesses. Simultaneously, such developments became publicly interpreted as examples and empirical proofs of "natural Roma (especially Roma women) susceptibility to smoking" or even to "vices in general".

3) Negative aspects of adherence

This kind regarded experiences of young Roma, especially in their teens and twenties, in their own understanding successfully experimenting with adherence in terms of health gains but finding some aspects of the adherence disappointingly negative. The expressions of disappointment were here often framed in racialized and gendered ethnic terms, and in contrast to local non-adherent practices, were considered as alternatives to the recommended adherence practices. E.g. allegedly clinically effective personal experiments with adherence to clinical recommendations regarding personal management of chronic diseases were often experienced as functional in terms of health benefits. However, they were also experienced as intrinsically connected with too substantial losses in terms of other, more important aspects of quality of life, such as: "unbearable" absences from "normal everyday affairs amid relatives", unbearable abstinences from favourite "pleasures", and feelings of loss of a "natural" Roma identity by becoming "too non-Roma like" (*gadžikano*).

4) Positive aspects of nonadherence.

This kind regarded Roma experiencing some aspects of their nonadherence (both deliberate and resulting from adherence failures) to specific recommendations and of their situation as significantly positive. Often, such positive experiences were framed in contrast to the respective adherent alternatives, and racialized and gendered ethnic terms were used. E.g. based on an experiment with both alternatives, one of the local extended families developed and held the view that detox and rehabilitation were not a reasonable therapy for alcohol-dependent persons in the settlement. Along with a comparable therapeutic effect, the "Roma way" alternative – taking care of such persons at home with attempts at controlled drinking – was namely experienced as earning substantial relative advantages for everybody involved (less family detachment, no personal estrangement, more personal care, happier patient, happier family, etc. Roma opinions differed here regarding whether such positives would be experienced as equally valuable for Roma and non-Roma due to supposed differences in their "nature" or "natural needs".

Appendix 3 Ethnographic vignettes exemplifying the most neglected of the identified local-level mechanisms supporting Roma nonadherence to clinical and public health recommendations

Roma socialization for their situation / Roma alternative practices / Roma self-exclusionary ideology and misinformation

D was a local young Roma woman (28) who married a non-local non-Roma truck driver and moved out of the settlement to live with him in a distant city. One day, a rumor spread in the settlement that after several years she is coming to visit her family. People from her extended family were expecting the visit with sincere excitement and curiosity, wondering "what she will look like after all these years" and "how she is doing for herself". On the day D arrived, she received a warm welcome from both local strangers, standing by in front of their homes, smiling and nodding kindly, as well as from her relatives, awaiting her with special meals prepared for this occasion, etc. Stepping out of her own "fancy car", D seemed nervous but happily moved. She then spent the whole day visiting the households of her closest relatives, talking, catching up with peoples' personal life stories. As a close relative of my (AB, first author) host, she also spent several hours in the household where I was staying. In the conversation, the youth in particular were curiously teasing out details about D's current way of life, asking how her work and household were, what friends did she have, how her marriage was, did she enjoy her personal freedom, etc. D was trying to portray her life as a happy one, although also politely stressing how she missed many aspects of the local life. The locals kept praising her achievement ("Oh, you brave girl, one can see you did really well for yourself!") and kept asking for ever more details. By the end of the day, D was being sent off with as kind a farewell as she had been welcomed with earlier. However, this time she was not able to hold her emotions and, as she was approaching her car, she burst into tears, and with her polite constant smile gone. This appeared to cause a general embarrassment, as most bystanders now hastily waved to her and hurried back to their homes.

Later in the evening, my host-mother's siblings sat together in her house for a regular evening meeting and discussion of the affairs of the passing day. I was struck with what followed: the locals started to talk about D and her life as if it was one big mistake. With local youth quietly and carefully listening to the adults' interpretations, as always, the adults kept reaching a general agreement that D, in the words of my host, "always tended to take things too far in the non-Roma direction (*gadžikano*), and now she is paying for it". In these interpretations, her present life was mostly sad: "Yes, she is working. But only as a helper in the local store, making ridiculous money. And there won't ever be a better job." "She spends most of her time alone, trapped in her nice apartment as in a prison, with no kids and her husband gone most of the time." To my question about the level of personal comfort and freedom she had achieved, i.e. something otherwise considered quite valuable locally, my host's sister replied: "Ok, so she has a shower now. But is she happy? No. Didn't you see the state of her when she was leaving?" Such interpretations of stories of segregated Roma switching to outside "non-Roma" standards were common and served locally as powerful, empirical arguments gradually adopted by local youth upon their own struggles with experiments with outside ways.

Drawbacks in adherence / Roma alternative practices / Roma self-exclusionary ideology and misinformation

M was a Roma man (50) suffering from alcohol dependency and an older brother of my (AB, first author) high-ranked host mother. During my first year of living in the settlement, M reached a very difficult phase of his sickness, as he lost the ability to take care of himself – several times a week his wife had to carry him home from the local pub. He was aggressive; he suffered from enuresis and encopresis; he had difficulties walking. At this stage, some of his younger siblings from high-ranked households decided to organize and pay the expenses connected with a rehab stay, and they convinced M's wife to cooperate "so our brother doesn't die". Over the next several months, the family kept spending valuable resources to pay weekly visits to M at a state facility located in a forest 70 km away. Each time we came to see M, both during the visits and in the car after we left, everybody tried hard to keep the mood up by stressing how much better M was: "Oh, you look twice as young as when they brought you here!" ("Did you see how he walks by himself again?") and constant joking. But there were many moments when everybody went silent, and it was palpable that people were struggling not to start crying instead. On several occasions, M couldn't hold it, and silently cried with his blue eyes wide open, fixating on his siblings. One day he formulated his silent reproach: "The people are not treating me badly here, no. And I get to talk a lot with this one nice non-Roma in my room. But still, I am all by myself here, you see?" After this visit and a quick passionate discussion in the settlement on the evening of our return, a month before the stay's planned ending, the siblings agreed resolutely with a sense of relief that they were "going to bring their brother back out of there!" In the week of M's return, his siblings organized a party to celebrate the reunion. The organizers made sure several bottles of M's favourite vodka were on the table, kept pouring him and he drank all of it. Over the next weeks, the family's hopes and plans were to supervise M's controlled drinking. In the words of my host: "A couple of beers a day will not turn him back to that state he was in." But they did, and over the next couple of months, everything returned to the state of affairs prior to the rehab.

This time, however, everybody was firm regarding that rehab was no longer an option. I was surprised and kept asking why, considering the rehab and abstinence obviously helped M to get back into shape physically and relieved his family, especially his wife, from tremendous everyday strain and struggle. A close friend, M's niece (25), somewhat angrily yet precisely summed up most of the answers I was getting: "Look, M is our uncle whom we love. He has always been very passionate about everything but was the nicest man to us before he got sick. It's your doctors who say drinking is a disease, right? And now that he is sick we should get rid of him, keep him locked up somewhere and deprive him of everybody he loves and of what he likes to do the most in the whole world!? Roma don't do this. Only you non-Roma have the hearts to do something like that to your close relatives. You should be ashamed of yourself! […] Everybody knows most people returning from rehabs will fall back to drinking. And this way, he can at least be taken care after by the people who love him. It's better for everybody!" This story and rationale well exemplify how, according to local preferences, many available recommended treatment venues for chronic diseases, especially where hospitalization required, were considered connected with too many drawbacks in terms of quality of life compared to local alternative approaches.
